# Limb Remote Ischemic Conditioning: Mechanisms, Anesthetics, and the Potential for Expanding Therapeutic Options

**DOI:** 10.3389/fneur.2018.00040

**Published:** 2018-02-06

**Authors:** Gangling Chen, Mrugesh Thakkar, Christopher Robinson, Sylvain Doré

**Affiliations:** ^1^Department of Anesthesiology, Center for Translational Research in Neurodegenerative Disease, University of Florida, Gainesville, FL, United States; ^2^Department of Pharmacology of Chinese Materia Medica, China Pharmaceutical University, Nanjing, China; ^3^McKnight Brain Institute, University of Florida, Gainesville, FL, United States; ^4^Department of Neurology, University of Florida, Gainesville, FL, United States; ^5^Department of Psychiatry, University of Florida, Gainesville, FL, United States; ^6^Department of Pharmaceutics, University of Florida, Gainesville, FL, United States; ^7^Department of Psychology, University of Florida, Gainesville, FL, United States; ^8^Department of Neuroscience, University of Florida, Gainesville, FL, United States

**Keywords:** ischemic conditioning, ischemic stroke, reperfusion injury, remote ischemic conditioning, stroke

## Abstract

Novel and innovative approaches are essential in developing new treatments and improving clinical outcomes in patients with ischemic stroke. Remote ischemic conditioning (RIC) is a series of mechanical interruptions in blood flow of a distal organ, following end organ reperfusion, shown to significantly reduce infarct size through inhibition of oxidation and inflammation. Ischemia/reperfusion (I/R) is what ultimately leads to the irreversible brain damage and clinical picture seen in stroke patients. There have been several reports and reviews about the potential of RIC in acute ischemic stroke; however, the focus here is a comprehensive look at the differences in the three types of RIC (remote pre-, per-, and postconditioning). There are some limited uses of preconditioning in acute ischemic stroke due to the unpredictability of the ischemic event; however, it does provide the identification of biomarkers for clinical studies. Remote limb per- and postconditioning offer a more promising treatment during patient care as they can be harnessed during or after the initial ischemic insult. Though further research is needed, it is imperative to discuss the importance of preclinical data in understanding the methods and mechanisms involved in RIC. This understanding will facilitate translation to a clinically feasible paradigm for use in the hospital setting.

## Introduction

When considered separately from other cerebrovascular diseases, ischemic stroke is ranked at number five among all causes of death; heart disease, cancer, chronic lower respiratory disease, and unintentional injuries are ranked ahead. In the United States, 795,000 strokes occur annually and 130,000 are fatal; that is, 1 of every 20 Americans is killed by stroke (1). Stroke is classified into two distinct categories: ischemic and hemorrhagic, for which ischemic accounts for approximately 87% (2, 3). During the initial reperfusion process following a transient ischemic attack (TIA), paradoxical injury occurs to the tissue distal to the site of the infarction. This process, known as reperfusion injury, is a direct consequence of downstream vascular restoration and tissue reoxygenation (4). Here, we review preclinical data with respect to reperfusion injury following ischemia in an attempt to translate such practice into clinical trials.

### Ischemic Stroke

In general, ischemic stroke occurs as a result of a clot, or thrombus, restricting distal blood flow at the site of occlusion. Multiple etiologies of ischemic stroke exist, including proximal cardioembolism, large artery atherosclerosis, and small vessel occlusion. From a molecular perspective, a main driver for brain metabolism is ATP consumption. ATP supply within the brain is dependent on continuous perfusion and, in scenarios of complete ischemia, approaches zero within about 4 min (5). This depletion of energy results in the activation of a cascade of molecular events ultimately leading to cell death (6, 7). The initial insult from ischemia results in an area of low perfusion surrounded by free radical formation and inflammation, with an overall preservation of structural integrity. This initial insult does not immediately correspond to irreversible damage, but as time progresses and the duration of ischemia lengthens, tissue infarction occurs and damage becomes irreversible. According to The National Institute of Neurological Disorders and Stroke Recombinant Tissue Plasminogen Activator (NINDS r-tPA) Stroke Study, early recognition of stroke symptoms and expeditious delivery of therapy decreases mortality and improves clinical outcomes (8). As the population continues to age, the incidence of ischemic stroke continues to rise due to a multitude of factors including (9, 10) hypertension (11, 12), diabetes (13–15), obesity (16), and metabolic syndrome (17). With the potential for stroke to become the number one cause of death in the United States, it is imperative that we continue to explore its pathologic mechanisms and pursue further research for alternative therapies.

### Ischemia/Reperfusion Injury

The clinical spectrum of stroke can vary widely. There is usually interdependency between the initial ischemic insult and the terminal completion of infarction. As mentioned above, during this interplay, there is also an intermediate step during reperfusion and after the initial ischemic event called ischemia/reperfusion (I/R) injury. Early during reperfusion, oxidative metabolism of arachidonic acid releases free radicals and generates nitric oxide (NO), which leads to peroxynitrite generation and lipid peroxidation (18). Acute or delayed cell death after I/R is what ultimately leads to the irreversible damage and the clinical sequelae seen in stroke patients (19). Delayed cellular death can be initiated by either internal events (intrinsic pathway through the mitochondria) or “death receptors” (extrinsic pathway) (20, 21). These molecular events occur in the area of infarction called the “ischemic penumbra.” Though rendered functionally silent due to the decrease in blood flow, the penumbra remains metabolically active throughout this process, leading to the activation of apoptosis-like processes hours to days after the initial ischemic event (22). Multiple molecular consequences exist in the setting of reperfusion, including the no-reflow phenomenon (23), production of oxygen free radicals (24), lipid peroxidation, activation of neutrophils, formation of arachidonic acid metabolites, stimulation of NO, and activation of endothelin. Collectively, these mechanisms lead to I/R injury. For example, in a study using feline model intestinal ischemia, Grace demonstrated that 4 h of ischemia alone resulted in less severe injury than 3 h of ischemia and 1 h of reperfusion (25).

### Ischemic Preconditioning and Postconditioning

Ischemic preconditioning is an endogenous mechanism of protection whereby short periods of sub-lethal ischemia performed in an organ confer protection against further ischemia in that same organ (26). Murry et al. found that transitory ischemia and reperfusion, prior to prolonged occlusion, reduces the injury of myocardial ischemia when compared to unconditioned occlusion controls (27). TIA, prior to a cerebral infarction, has been shown in multiple studies to confer neuroprotection by decreasing the size of infarction and improving neurological outcomes (28). Extensive research shows that ischemic preconditioning treatment reduces cerebral damage (29–31); however, its use in the clinical setting has been limited due to the unpredictable nature of cerebral infarctions. Understanding the underlying mechanisms of how ischemic preconditioning offers protection against stroke-induced neuronal death is imperative for translation into medical practice.

Ischemic postconditioning is a process, following reperfusion of a vessel, in which transient episodes of ischemia are induced so as to limit reperfusion injury. This process stimulates protective factors thereby limiting inflammation and delayed cell death (31, 32). Studies using experimental animal models have shown that postconditioning reduces myocardial I/R injury and proved its protective molecular functions (33–35). Evidence of postconditioning in cerebral ischemia has been thus far limited to preclinical studies.

Zhao and colleagues, using canine animals in a myocardial study, conducted occlusion of the left anterior descending artery (LAD) for 60 min, followed by reperfusion for 3 h (36). Three cycles of 30-s reperfusion and 30-s LAD re-occlusion preceded the 3 h of reperfusion. The myocardial infarct size was reduced significantly in the postconditioning group compared with the control group.

Hence, these studies suggest that postconditioning blocks TUNEL-positive cells (apoptotic-like cells) in the penumbra, thereby reducing cell death and reducing oxygen free radical formation after infarction (36). Thus, postconditioning should be considered as a possible future therapeutic target as it reduces the severity of infarction in both myocardial and cerebral models.

### Limb Remote Ischemic Pre-, Per-, and Postconditioning

**Figure 1:**
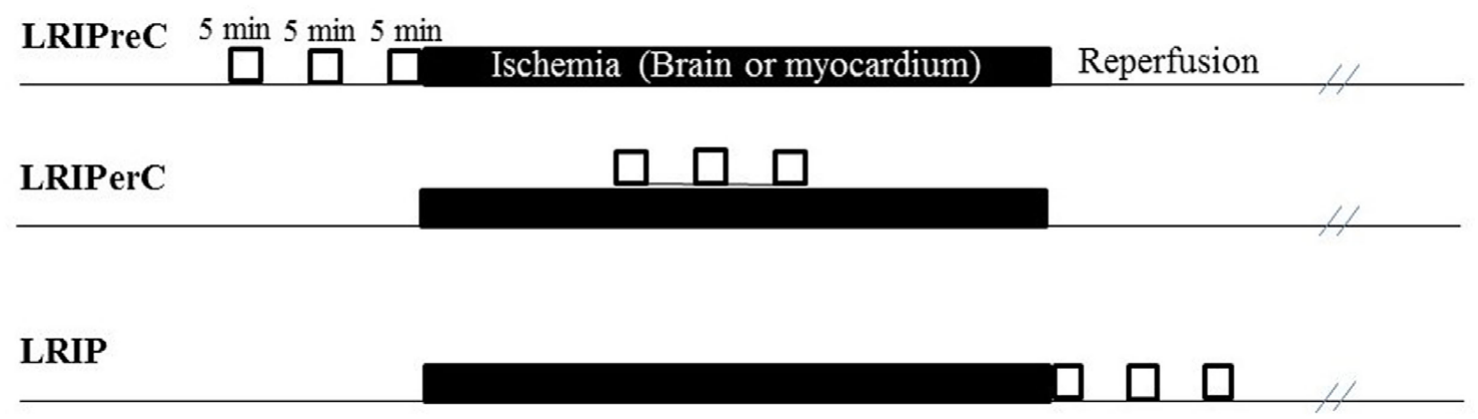
Visual representation showing temporal relationship between limb remote ischemic preconditioning (LRIpreC), limb remote ischemic preconditioning (LRIperC), and limb remote ischemic postconditioning (LRIP) and the ischemic insult and reperfusion.

The aforementioned concepts of ischemic preconditioning and postconditioning relate to the modulation of vascular hydrodynamics within a single ischemic organ. These terms have been broadened to include that of limb “remote” preconditioning, perconditioning, and postconditioning. In remote pre-, per-, or postconditioning, a non-vital, non-ischemic organ will undergo reversible, interrupted occlusion and reperfusion of arterial flow before or after a vital organ endures ischemia (26, 37, 38). The temporal relationship between limb remote ischemic preconditioning (LRIpreC), limb remote ischemic perconditioning (LRIperC), and limb remote ischemic postconditioning (LRIP) and the ischemic insult and reperfusion are shown in Figure 1.

More recently, studies of cerebral ischemia have shown attenuation of cerebral I/R injury. Limb remote ischemic preconditioning (LRIpreC) is defined as inducible ischemia of a hind limb that confers protection to the brain from subsequent ischemic injury (39). Research shows that LRIpreC is able to confer neuroprotection via temporarily reducing vascular perfusion to the brain (40). Ren et al. were the first to show that LRIpreC reduced infarct size in a rat model (41). Further, studies have failed to verify a single molecular pathway as vital, but both humoral and neural mechanisms provide protection. The clinical counterpart of LRIpreC in ischemic stroke would be TIA.

Limb remote ischemic perconditioning (LRIperC), defined as conditioning during an active incidence of ischemia, offers a potential treatment. The first study of LRIperC by Schmidt et al. in 2006, using a pig model (42), demonstrated that four cycles of 5-min of hind limb ischemia followed with 5-min of perfusion reduced myocardial infarct size.

Limb remote ischemic postconditioning (LRIP) is a process in which transient I/R is applied to a non-vital remote organ following reperfusion of previously ischemic organ (43). LRIP has been shown to attenuate the effects of I/R injury; however, the mechanisms are still not clear.

## Mechanisms of Limb Remote Ischemic Pre-, Per-, and Postconditioning

The communication and transmission of the protective effect between the remote ischemic-reperfused vessels to the brain is complex and involves multiple pathways. Clinical studies have shown there is a strong indication that remote ischemic conditioning (RIC) reduces I/R injury (44–47); however, the mechanisms of RIC in clinical studies are still being studied. There have been multiple preclinical studies done in animal models, specifically rats, of LRIpreC (26, 38, 41, 48–53). These studies have collectively shown that LRIpreC performed prior to the onset of cerebral ischemia reduced the size of infarction by an average of 80% at 48 h (54). The use of remote postconditioning in cerebral ischemia, by instituting occlusion of the femoral artery during the time of reperfusion, showed a 67% reduction in infarct volume 48 h following middle cerebral artery occlusion (MCAO) (26). There are numerous mechanisms to account for the reduction in cerebral infarct size. Thus, the purpose of this review is to discuss these purported mechanisms to understand the physiology and pathology for translation to clinical studies. An extensive list of known studies and their descriptions on LRIpreC, LRIperC, and LRIP can be found in Table 1.

**Table 1:** Summarized description of reported preclinical studies of limb remote preconditioning, limb remote perconditioning, and limb remote postconditioning.

	Animal	Sex, age, body weight	Ischemic organ	Anesthetic used prior to ischemia	Anesthetic used during RIC	RIC protocol/day	When RIC was started	RIC organ	Main pathway investigated	Reference
Limb remote ischemic postconditioning (LRIP)	C57BL/6 mice	Male, 10 ± 1 weeks	Brain	Isoflurane; dose not mentioned	Not mentioned	3 cycles, 5 min ischemia and 5 min reperfusion/day for 2 weeks	At 1 week after bilateral common carotid artery stenosis	Bilateral hind limb	Inflammatory responses and Aβ accumulation, ICAM-1, VCAM-1 were detected	(55)
	C57BL/6 mice	Male, 10 weeks	Hind limb	Pentobarbital 60 mg/kg	Not mentioned	2 cycles, 5 min/day for 1 day	At the beginning of reperfusion	Left hind limb	Endogenous adenosine receptor	(56)
	CD1 mice	Male, adult, 25–30 g	Brain	10% Chloral hydrate	Not mentioned	3 cycles, 5 min/day for 1 day	At the beginning of reperfusion	Femoral artery of bilateral hind limbs	Nrf2-ARE pathway	(52)
	C57BL/6 mice	Male, 8–12 weeks	Heart	4% Isoflurane and maintained with 1–2%	Not mentioned	3 cycles, 5 min/day for 1 day	At the beginning of reperfusion	Left limb	Autophagy	(57)
	C57BL/6 mice	Male, 6–8 weeks, 25–30 g	Limb	Pentobarbital 60 mg/kg	Pentobarbital 60 mg/kg	1 cycle, 2 h ischemia and 24 h reperfusion	At 0 min, 20 min, or 2 h after left leg ischemia	Right leg	No specific pathway mentioned	(58)
	C57BL/6 mice	Male, 10 weeks, 23–25 g	Limb	Pentobarbital 60 mg/kg	Not mentioned	2 cycles, 5 min/day for 1 day	At 20 min before the reperfusion of the right hind limb	Left leg	Activation of adenosine receptors	(38)
	B6129SF2/J F2 mice	Male and female equally among groups, 10 and 16 weeks	Heart	Pentobarbital 90 mg/kg	Not mentioned	Remote trauma (abdominal incision) and have termed this phenomenon remote preconditioning of trauma	At the beginning of reperfusion	Coronary artery	Bradykinin, β-adrenergic receptors, and PKC signaling pathways	(59)
	C57BL/6 mice	Male, 8–10 weeks	Heart	Pentobarbital 60 mg/kg	Pentobarbital 60 mg/kg	4 cycles, 5 min/day (Acute rIPC 1 time, Delayed rIPC one time, Chronic rIPC 9 times/9 days)	At 20 min before ischemia	Femoral artery	Downregulation of mTOR and enhanced autophagy signaling	(60)
	Sprague-Dawley (SD) rats	Male, 300–320 g	Brain	1.75% Isoflurane and maintained with 1–2%	Not mentioned	3 cycles, 10 min bilateral femoral artery	At 0 min, 10 min, or 30 min after reperfusion start	Bilateral femoral artery	AKT/GSK3β-dependent autophagy	(48)
	SD rats	Female, 250–300 g	Heart	10% Chloral hydrate	Not mentioned	3 cycles, 30 s/day 1 day	At the beginning of reperfusion	Limb	Mainly attributed to hospitable milieu for engrafted cells	(49)
	SD rats	Male, 290–310 g	Brain	Pentobarbital 50 mg/kg	0.25% Bupivacaine hydrochloride	2–3 cycles, 15 min; 3 cycles, 5 min/day for 1 day	At 3 or 6 h after reperfusion	Bilateral femoral artery	Through opening of *K*_ATP_ channels	(61)
	SD rats	Male, 270–330 g	Brain	5% Isoflurane and maintained with 1–2%	1–2% Isoflurane	3 cycles, 15 min/day for 1 day	At the beginning of reperfusion or 3 or 6 h after reperfusion	Left limb	Protein synthesis inhibitor and nerve blocker eliminate the protective effect of rapid LRIP	(26)
	SD rats	Male, 250–280 g	Brain	3.6% Chloral hydrate	Not mentioned	3 cycles, 10 min/day for 1 day	At the beginning of reperfusion	Proximal hind limbs of each rat	HIF1α	(62)
	SD rats	Male, 300–320 g	Brain	10% Chloral hydrate	Not mentioned	3 cycles, 10 min/day for 1 day	At the beginning of reperfusion	Bilateral femoral artery	AKT pathway	(63)
	SD rats	Male, 290–350 g	Skin flap reperfusion injury model	Ketamine 80 mg/kg	Not mentioned	Group1:3 cycles, 5 min/day for 1 day; Group2:1 cycle, 15 min/day for 1 day	At the beginning of reperfusion	Right hind limb	Attenuation of oxidative stress. For Group 1 and 2, malondialdehyde content lower, superoxide dismutase activity and flap survival rates higher than control group. The effects of LRIP for Group 1 were better	(64)
	SD rats	Male, 200–250 g	Brain	10% Chloral hydrate	Not mentioned	3 cycles, 15 min/day for 1 day	Immediately after 8 min of 4-VO	Bilateral hind limbs	PI3K/Akt pathway	(65)
	SD rats	Male, 250–280 g	Brain	Chloral hydrate 330 mg/kg	Not mentioned	3 cycles, 5 min/day for 1 day	At 0, 1, and 3 h after reperfusion	Left femoral artery	MyD88-TRAF6-P38 MAP-kinase pathway of neutrophils	(50)
	SD rats	Unsexed pups, 10 days	Brain	3% Isoflurane and maintained with 1.5–2%	Not mentioned	4 cycles, 10 min/day for 3 days	At 24 h after HI surgery	Hind limb	No specific pathway mentioned	(66)
	SD rats	Male, 250–300 g	Brain	Chloral Hydrate 300 mg/kg	Not mentioned	3 cycles, 5 min/day for 1 day	At the beginning of reperfusion	Right hind limb	Upregulating STAT3 and reducing apoptosis	(67)
	SD rats	Male, 250–300 g	Retinae	1.5–3.5% Enflurane and maintained with sodium pentobarbital 30 mg/kg	Sodium Pentobarbital 30 mg/kg	3 cycles, 10 min/day for 6 days	Began on the second postoperative day after retinal ischemic injury.	Hind limb	Through the upregulation of antioxidative stress proteins, such as Nrf2 and heme oxygenase 1	(68)
	SD rats	Male, 330–380 g	Brain	10% Chloral hydrate	Not mentioned	3 cycles, 15 min/day for 1 day	At the same time as reperfusion	Left femoral artery	By reversing endothelial nitric oxide synthase uncoupling	(43)
	SD rats	Male, 280–320 g	Brain, intracerebral hemorrhage	1.5% Halothane and maintained with 1.5%	Not mentioned	3 cycles, 10 min/day for 1 day	At 1 h after collagenase injection	Bilateral femoral arteries	Role of AQP4 and MMP9	(69)
	SD rats	Male, 290–330 g	Brain	4% Enflurane and maintained with 2%	Not mentioned	4 cycles, 10 min/day for 1 day	At the same time as the bilateral common carotid arteries reperfusion	Bilateral femoral arteries	Peripheral nerves	(70)
	SD rats	Male, 250–280 g	Heart	Pentobarbital 80 mg/kg	Pentobarbital 30 mg/kg	4 cycles, 5 min/day for 1 day	At the same time as reperfusion; intermittently (every 3 days) and intensively (every day), both for 28 days	Hind limb	Oxidative stress, inflammatory cell migration	(71)
	Wistar rats	Male, 250–350 g	Heart	Pentobarbital 50 mg/kg	Not mentioned	3 cycles, 30 s	At the same time as reperfusion	Coronary occlusion	PI3K/Akt-dependent cell-survival signaling	(72)
	SD rats	Sex not mentioned, 10 days	Brain	3.5% Isoflurane and maintained with 1–2%	Not mentioned	4 cycles, 10 min/day for 1 day	At immediately after neonatal hypoxia–ischemia	Hind limb	Activation of the opioid receptor/PI3K/Akt signaling pathway	(73)
	SD rats	Male, 250–350 g	Heart	10% Chloral Hydrate	Not mentioned	3 cycles, 10 min/day for 1 day	At the same time as reperfusion	Ischemic cardiac grafts	Heat shock protein 70 and oxygen radical absorbing capacity	(74)
	SD rats	Male, 250–350 g	Heart,	Pentobarbital 80 mg/kg	Pentobarbital 20–40 mg/kg	1 cycle, occluded for 5 min and released for 1 min/day for 1 day	At the same time as reperfusion	Renal artery	Adenosine receptors	(75)
	SD rats	Male, 300–320 g	Brain	1.75% Isoflurane	Not mentioned	3 cycles, 10 min/day for 1 day	At 30 min of ischemia, RIC at the onset of reperfusion	Femoral artery	AKT pathway	(76)
	SD rats	Male, 250–280 g	Brain	3.6% Chloral Hydrate	Not mentioned	3 cycles, 10 min/day for 1 day	At the same time as reperfusion	Proximal hind limbs	HIF1α	(62)
	Wistar rats	Male, 12–15 weeks, 270–300 g	Liver, kidney	Ketamine 70 mg/kg and Xylazine 10 mg/kg	Not mentioned	3 cycles, 5 min/day for 1 day	At 10 min or 60 min after the end of the LRIP the liver and kidney were harvested for biochemical analysis.	Left hind limb	Improves antioxidant defense	(77)
	SD rats	Female, 7 weeks, 250–280 g	Brain	10% Chloral Hydrate	Not mentioned	3 cycles, 10 min/day for 1 day	At the same time as reperfusion	Bilateral femoral arteries	AQP4 downregulation in astrocytes	(78)
	SD rats	Male, 250–300 g	Heart	Not mentioned	Not mentioned	3 cycles, 5 min/day for 1 day	At the same time as reperfusion	Bilateral femoral arteries	Transient Receptor Potential Vanilloid 1 (TRPV1) activation	(79)
	SD rats	Male, 8–10 weeks, 220–280 g	Brain	10% Chloral Hydrate	Not mentioned	3 cycles, 10 min/day for 1 day	At the same time as reperfusion	Bilateral femoral arteries	p38MAPK signal pathway	(80)
	SD rats	Male, 9 weeks old, 290–320 g	Heart	Pentobarbital 60 mg/kg	Not mentioned	1 cycle, 10 min ischemia, 5 min reperfusion/day for 1 day	At 15 min after left coronary arteryligation, and limb blood supply was restored 5 min before loosening the left coronary artery ligation for myocardial reperfusion.	Two hind limbs	κ–opioid receptors	(81)
	SD rats	Unsexed pups, 10 days	Brain	3% Isoflurane and maintained with 1.5%	1.5% Isoflurane	4 cycles, 10 min/day for 1 day	At 24 h after hypoxia ischemia.	Hind limb	No specific pathway mentioned	(66)
Limb remote ischemic preconditioning (LRIpreC)	C57BL/6 mice	Male, 10 days old	Heart	3% Isoflurane and maintained with 1.5%	Pentobarbital 60 mg/kg	4 cycles, 5 min/day for 1 day	Heart ischemia was induced immediately after LRIpreC	Hind limb	By systemic releasing of microRNA 144	(82)
	C57BL/6 mice	Male, 9–12 weeks	Heart	Pentobarbital 70 mg/kg	Not mentioned	3 cycles, 5 min/day for 1 day	At 24 h before ischemia	Left femoral artery	By upregulating IL10	(83)
	SD rats	Male, 250–350 g	Brain	5% Isoflurane and maintained with 2–3%	2–3% Isoflurane	3 cycles, 15 min/day for 1 day	Brain ischemia was induced immediately after LRIpreC	Left hind limb	No specific pathway mentioned	(84)
	SD rats	Male, 280–320	Brain	4% Isoflurane and maintained with 2%	2% Isoflurane	3 cycles, 5 min/day for 1 day	Brain ischemia was induced at 1 h after LRIpreC	Right hind limb	Depend on the activation of adenosine A1 receptors and by reduction in oxidative stress, inflammation and endogenous antioxidant preservation	(85)
	Wistar rats	Male, 250–280 g	Brain	10% Chloral Hydrate	Not mentioned	3 cycles 5 min; 3 sessions/day for 3 days	Brain ischemia was induced immediately after the last LRIpreC operation	Left common carotid artery	No specific pathway mentioned	(86)
	SD rats	Male, 270–330 g	Brain	5% Isoflurane and maintained with 1–2%	1–2% Isoflurane	2–3 cycles, 5 or 15 min (2 cycles, 5 min; 3 cycles, 5 min; 2 cycles 15 min, 3 cycles 15 min)/day for 1 day	Brain ischemia was induced immediately or 12 h, 2 d after LRIpreC	Left hind limb	No specific pathway mentioned	(41)
	Wistar rats	Male, 240–260 g	Heart	Urethane 1 g/kg	Chloral Hydrate 0.3 g/kg	3 cycles, 5 min/day for 3 days	At day 4	Left hind limb	Decreased leakage of myocardial enzymes	(87)
	SD rats	Male, 280–320 g	Brain	5% Isoflurane and maintained with 2.5–3%	2.5% Isoflurane	4 cycles, 5 min/day for 1 day	At 1 h before middle cerebral artery occlusion (MCAO)	Bilateral hind limb	Significant alterations in peripheral immune responses	(88)
	Wistar rats	Unsexed pups, 150–200 g	Heart	Thiopental 35 mg/kg	Not mentioned	4 cycles, 5 min/day for 1 day	At 10 min before the heart ischemia	Hind limb	TRPV1 channels	(89)
	Wistar rats	Male, 8–10 weeks	Limb	Pentobarbital 60 mg/kg	Not mentioned	10 min limb ischemia followed by 5 min (LRIpreC5) or 10 min (LRIpreC10) reperfusion/day for 1 day	Collected blood sample at 5 min (LRIpreC5) or 10 min (LRIpreC10) after limb ischemia	Right femoral artery	Effect on plasma proteome	(90)
	SD rats	Male, 270–330 g	Brain	5% Isoflurane and maintained with 1–2%	1–2% Isoflurane	3 cycles, 15 min/day for 1 day	At 1.5 h before dMCAO	Left femoral artery	Extrinsic apoptotic pathway and TNF-related apoptosis-inducing ligand receptors expression	(40)
	Wistar rats	Male, 150–200 g	Heart	Thiopental 35 mg/kg	Not mentioned	5 cycles, 5 min/day for 1 day	Heart ischemia was induced immediately after LRIpreC	Hind limb	Activation of mechanosensitive TRP and especially TRPV channels	(91)
	SD rats	Male, 280–320 g	Heart	Pentobarbital 60 mg/kg	Pentobarbital, 10–15 mg/kg	15 min occlusion followed by 10 min reperfusion/day for 1 day	Heart ischemia was induced immediately after LRIpreC	Both hind limbs	Circulating factors released by visceral organs	(92)
Limb remote ischemic per-conditioning (LRIperC)	C57BL/6J mice, ovariectomized	Female, 20 ± 2 weeks	Brain	Mild Isoflurane; dose not mentioned	Not mentioned	4 cycles, 10 min/day for 1 day	At 2 h poststroke	Limb	No specific pathway mentioned	(53)
	C57BL/6J mice	Male, 20 ± 1 weeks	Brain	3.5% isoflurane and maintained with 1.5 – 2.0%	Not mentioned	5 cycles, 5 min/day for 1 day	At 2 h after embolic MCAO	Left limb	No specific pathway mentioned	(93)
	SD rats	Male, Postnatal day 60	Brain	Ketamine Hydrochloride 80–100 mg/kg and Acepromazine Maleate 5 mg/kg	Not mentioned	4 cycles, 5 min/day for 1 day	At 40 min prior to MCAO	Left hind limb		(94)
	SD rats	Male, 250–280 g	Brain	10% Chloral Hydrate	Not mentioned	4 cycles, 5 min/day for 1 day	At 40 min prior to reperfusion	Left hind limb	Inhibits autophagy to attenuate plasma high mobility group box 1 and induce neuroprotection	(51)
	SD rats	Male, 280–320 g	Brain	10% Chloral Hydrate	Not mentioned	4 cycles, 10 min/day for 1 day	At the start of 10 min after MCAO	Bilateral femoral arteries	Activation of autophagy/lysosomal pathway	(95)
	Wistar rats	Male, 240–290 g	Kidney	5% sevoflurane and maintained with 2.5–3%	Not mentioned	4 cycles, 5 min/day for 1 day	At 40 min before kidney ischemia (LRIpreC) or during (LRIperC) by clamping the infrarenal aorta	Part of the abdominal aorta just above the aortic bifurcation	Associated with pAkt and pERK1/2 upregulation and increased HSP expression	(96)

### Oxidative Stress

Evolving evidence has shown that oxidative stress is vital in the pathophysiologic mechanism of cerebral I/R injury. The buildup of excessive free radicals leads to oxidative damage on proteins, lipids, and nucleic acids, which subsequently leads to the progression of ischemic injury after stroke (52, 97–99). Of interest, we have showed that Nrf2, a transcription factor that regulates the expression of antioxidant proteins, attenuated tissue injury in the cerebral ischemia model in mice (100, 101). When the body falls under oxidative stress, Nrf2 translocates from the cytosol to the nucleus and binds to a DNA promoter to initiate transcription of antioxidative genes (102). This promoter antioxidant response element (ARE) regulates the expression of antioxidant enzymes and proteins.

For LRIpreC, Wei and colleagues used rats following myocardial infarction to study the effects of LRIpreC versus perconditioning and postconditioning and the role of malondialdehyde (MDA) in oxidative pathways (71). They used four cycles of 5-min hindlimb ischemia with subsequent 5-min reperfusion; this was done every 3 days for a total of 28 days. The results showed that MDA was increased in control rats and was reduced by per- and postconditioning in a dose-dependent fashion (71). Jia et al. tested the role of MDA and myeloperoxidase (MPO) in rats that underwent liver transplantation. Results of their study showed that the production of MDA and MPO was markedly reduced in the LRIpreC group (103).

For LRIperC, Costa et al. used combined LRIperC and local postconditioning in rats that underwent 60 min of liver ischemia (104). The procedure consisted of four cycles of 5-min hind limb ischemia and 5-min perfusion; local postconditioning consisted of four cycles of 5-min liver ischemia followed by 5-min perfusion. Results showed that the combination of LRIperC and local postconditioning was able to reduce hepatic tissue MDA levels and further attenuate I/R injury (104).

For LRIP, Li et al. used CD1 mice to prove that LRIP could significantly reduce the I/R injury through upregulation and expression of Nrf2 along with heme oxygenase 1 (HO1), quinone oxidoreductase 1 (NQO1), and superoxide dismutase (SOD), all cytoprotective enzymes downstream of Nrf2 (52). Their group used mice to conduct three cycles of 5-min ischemia and subsequent 5-min reperfusion of bilateral femoral arteries to show that LRIP significantly improved neurological outcomes likely by reducing oxidative stress and initiating the Nrf2-ARE pathway. Zhang et al., Zhou et al., and Kadkhodaee et al. all investigated the effect of LRIP against I/R injury in rats; all groups showed a significant decrease in the level of MDA after LRIP (64, 105, 106). We performed studies in rats to understand the role of nitrotyrosine, mRNA of P22^phox^, and xanthine oxidase and how they contribute to oxidative damage. During three cycles of 15-min occlusion and subsequent 15-min reperfusion of the left femoral artery, the levels of these three oxidants were decreased by LRIP. Further experimentation proved that LRIP could reverse the eNOS uncoupling to reduce the I/R injury caused by the aforementioned oxidants (43).

Other researchers also proved that RIC was able to reduce the oxidative stress under I/R injury. Oliveira et al. studied RIC in the kidney model in rats to further understand the role of MDA (107). Their studies showed similar results in which RIC was able to reduce the level of MDA and further attenuate I/R injury (107). Silva et al. studied the relationship between RIC and antioxidant activity in rats, and their experiment consisted of three cycles of 5-min left hind limb ischemia followed by 5-min perfusion (77). Their results showed that RIC increased both liver and kidney antioxidant capacity after 10 min; there was no difference seen at the 60-min time interval (77). Zhang et al. researched the effects of pro-inflammatory proteins iNOS and nuclear factor kappa B (NFκB) and how they are affected by LRIperC and LRIP (108). They used APAP (acetaminophen/oxycodone) with mice and performed LRIperC and LRIP, which showed substantially decreased APAP-induced serum levels of ALT, AST, Tumor Necrosis Factor α (TNFα), IL-6, hepatic MDA, and nitrotyrosine formation, proving the antioxidative effects remote conditioning has. It also improved hepatic SOD, GSH, and glutathione peroxidase activities to facilitate protection from I/R injury. Thus, both LRIperC and LRIP effects were twofold in a sense that they prevented the damage from oxidative proteins and upregulated antioxidative proteins to increase protection (108).

### Inflammatory Changes

Limb remote ischemic preconditioning and LRIP have been shown to protect against I/R injury by downregulating the key steps leading to systemic inflammation. Reports have shown this process blocks NFκB, subsequently reducing systemic inflammation. Kim et al. showed that LRIP had protective effects on lipopolysaccharide (LPS)-induced systemic inflammation (109). Their group induced three cycles of 10-min ischemia followed by 10-min reperfusion cycles to right hind limbs before and after LPS injection. The results indicated the survival rate within 120 h was increased in the LPS-injected and remote postconditioning mice. NFκB activation was suppressed and HO1 levels were markedly elevated in the LPS-injected and remote postconditioned mice. This ultimately led to decreased neutrophil infiltration and a decreased systemic inflammatory response. Hess et al. worked with mice to show that bilateral carotid artery stenosis triggered a pro-inflammatory milieu and augmented gene expression of ICAM-1 and VCAM-1 (55). ICAM-1 and VCAM-1 promote adhesion directly causing the disintegration of the blood–brain barrier (BBB) and resulting in increased infiltration of pro-inflammatory immune cells. When these mice were treated with LRIP, there was a reduced vascular inflammatory response. Redington et al. studied chemokines and their role in selectively recruiting monocytes, neutrophils, and lymphocytes leading to the inflammatory response (71). Specifically, they looked at monocyte chemoattractant protein-1 (MCP-1) and its responsibility for the induction of monocytes in inflammatory changes. Through RIC, they were able to downregulate pro-inflammatory pathways and notice a decline in MCP-1 leading to less damage and improved post-MI recovery. Interleukins also play an essential role in the inflammatory process after both a myocardial infarction and stroke. Okano et al. investigated IL-6 and how its expression increases in the acute phase of cerebral ischemia (110). They used an anti-mouse IL-6 receptor monoclonal antibody to block IL-6 signaling. At 24 h after MCAO, blockade of IL-6 caused an increased number of apoptotic cells and a subsequently larger infarct size and thus concluded that endogenous IL-6 played an important role in preventing damaged neurons from undergoing cell death. Adenosine is also involved in the inflammatory process and is discussed in detail below.

### Cerebral Edema

Cerebral edema can be a life-threatening, consequential condition that develops secondary to a pro-inflammatory state; it occurs after a cerebral infarction. Edema ensues in response to cellular swelling, breakdown of the BBB (increasing cellular permeability), and/or increased osmotic pressure from the leakage of cellular products. Cerebral edema can be categorized into four separate categories: vasogenic, cytotoxic, osmotic, and interstitial. Cytotoxic, dysfunction in the sodium and potassium pump, and vasogenic, disruption of the BBB integrity, are the two most common types of edema that ensue during cerebral infarction (78). Thus, it is important to focus on a protein called aquaporin (AQP) that plays a vital role in the pathogenesis of cerebral edema. More specifically, AQP4 is a water channel protein located in the end foot of astrocytes; these molecules become upregulated when a cerebral insult occurs (78). Previously, it has been shown that AQP4 could be related to the increased permeability of the BBB in cerebral I/R injury; it allows this by enhancing transmembrane water flux in astrocytes (78). Thus, AQP4 dysregulation resulting in astrocyte swelling is representative of cytotoxic edema. Cerebral edema, from the over expression of AQP4, is deemed to be a key determinant in neuronal damage during cerebral ischemia, though the role of this molecule in RIC is still unknown.

Another molecule involved in the formation of cerebral edema is Matrix metallopeptidase 9 (MMP-9). MMP-9 is an intracellular protease that degrades components of the tight junctions between the endothelial cells, thereby allowing disruption of the BBB (69). Furthermore, this disruption of the BBB allows for the free flow of water into the extracellular space of the brain leading to increasing cerebral edema. MMP-9’s pathogenesis of cerebral edema falls under the classification of vasogenic edema (78).

Performing LRIP using three cycles of 10-min ischemia and 10-min perfusion in hind limbs, Li et al. used female rats to test the neuroprotective effect of LRIP in ischemic stroke models and determine the protective mechanisms of AQP4 (78). Results showed decreased cerebral infarct size, edema, and BBB disruption, and overall improved functional neurologic recovery following stroke via downregulation of AQP4 in astrocytes. Zong et al. induced MCAO in Sprague Dawley (SD) rats to show the relationship between LRIP and cerebral edema (62). Ischemia was performed for a total of 60 min; three cycles of 10-min occlusion followed by 10-min perfusion were done. Results were promising and showed dramatically reduced cerebral edema in LRIP-administered rats (62).

Performing LRIP using three cycles of 5-min occlusion followed by 5-min reperfusion in bilateral femoral arteries, Li et al. used CD1 mice to induce MCAO and study the effects LRIP had on cerebral edema (52). They found that LRIP significantly improved neurological outcomes by reducing infarct size and decreasing brain edema (52). Liu et al. also used SD rats to induce MCAO to study the effects of LRIP had on cerebral edema. Results showed improved neurological outcomes by reducing infarct size and decreasing brain edema (111).

### Hemodynamic Sequela

The circulatory system is controlled by a rather sensitive, homeostatic mechanism in the body that continuously monitors and adjusts to changes from equilibrium in the body. These changes are seen in the hemodynamics, or blood flow, in the body and thus can affect a person’s recovery or outcome from ischemic stroke. Endothelial nitric oxide synthase (eNOS) is a protein accountable for the production of endothelium-derived NO and is involved heavily in cerebral I/R injury. NO is a potent vasodilator and plays a critical role in equilibrating blood pressure and overall hemodynamics in the body. Thus, it has been hypothesized that enhancing NO availability would markedly improve microcirculation and antagonize I/R injury by reducing the production of ROS (112). He et al. evaluated the protective effects of both LRIperC and LRIP after liver transplantation in rats (112). The grafts subjected to LRIperC showed significant improvement in both hepatic and remote organ function; there was no significant difference between LRIperC and LRIP grafts. Thus, they were able to show that liver graft protection of LRIperC involved the inhibition of ROS and the upregulation of the eNOS/NO pathway. Additionally, increased expression of heat shock protein 70 (HSP70) is seen in the ischemic penumbra, signifying its role in the attenuation and protection of ischemia. Dubey et al., using cerebral ischemia mice models, showed overexpressing HSP70 protected against myocardial and cerebral ischemia (MCA occlusion); preconditioning with ischemia showed enhanced expression of HSP70 in the cell (113).

### Cellular Death

Neuronal cell death is well known to have a prominent role in the progression of brain damage in ischemic cerebral stroke. Thus, as an area of focus, it is important to discuss the clinical relevance of studying anti-cell death and its translation from preclinical studies to clinical ones. The pathways of neuronal cell death can be complex; however, it is important to focus on a pro-apoptotic member of the TNF family called TNF-related apoptosis-inducing ligand (TRAIL). This ligand is released by glia, injured neurons, and leukocytes. TRAIL has been shown to mediate neuronal apoptosis through binding on its receptors post cerebral ischemia (40). Xu et al. used male SD rats to induce LRIperC using three cycles of 15-min left hind limb ischemia followed by 15-min reperfusion (40). TUNEL staining and cleaved caspase-3 expression indicated that ischemia-induced neuronal apoptosis was attenuated. In addition, LRIperC might partially suppress TRAIL-activated extrinsic apoptosis through downregulation of TRAIL death receptors and upregulation of TRAIL decoy receptors.

Signal transducer and activator of transcription 3 (STAT3) is a protein that carries stress signals from the plasma membrane to the nucleus (114). It has been shown that STAT3 is involved in I/R injury by binding to a STAT target site that becomes enhanced after the initial insult. This protection was initially discovered and described in mice with a cardiac-specific deletion of STAT3; which showed an increased infarct size compared to those mice that had active STAT3 (114). In the nervous system, STAT3 is involved in the government of cellular apoptosis. Thus, decreased levels of STAT3 translated to a decreased protective effect from an ischemic insult. Cheng et al. induced MCAO in rats, and LRIP was performed on the right hind limb for three cycles of 5-min ischemia and 5-min reperfusion (67). Their results showed the protein expression of phosphorylated STAT3 was increased in the LRIP group as opposed to the control group. This further indicates that activation of STAT3 facilitates the attenuation of neuronal apoptosis and inflammation.

Bax is a protein in the Bcl-2 gene family that regulates apoptosis. Studies have shown increased transcription of Bax during ischemic insults that result in increased cellular death and necrosis. Thus, multiple studies have demonstrated the effect LRIP has on the level of proapoptotic proteins Bax and caspase-3. Results showed when either LRIperC or LRIP was applied there was a reduction in the expression of caspase-3 and Bax, effectively decreasing apoptosis. This reduction showed a decreased incidence of I/R injury after initial ischemic insult. These studies were done in rats in both cerebral and myocardial models (65, 70, 115–120).

Bradykinin has also shown to be involved in ischemic preconditioning, ischemic postconditioning, and remote conditioning as an anti-apoptotic agent by acting as an endogenous, cytoprotective mediator in ischemic tissue. Sharma et al. showed that bradykinin confers its protection via activation of the PI3K/Akt/eNOS signaling pathway and regulation of redox state via NO release (121). During postconditioning, they showed that bradykinin confers neuroprotection mainly through augmented redox signaling and activation of the mitochondrial anti-apoptotic pathway. Hence, during remote conditioning, the activation of B_2_ receptors results in the configuration of signalosomes that activate intracellular cytoprotective transduction pathways.

### Autophagy

Autophagy is a natural, destructive mechanism that degrades and recycles cellular components; it also disassembles and removes any dysfunctional cellular components. Recent evidence has shown the protective role that autophagy plays in I/R injury. It does so by consuming damaged and dysfunctional mitochondria to counteract the release of cytochrome C and death signaling (122). HO1 is a protein that has been studied for its properties to limit inflammation and prevent cell death. Wang et al. studied the relationship between HO1 and autophagy by inducing hepatic I/R injury in male mice (122). LRIpreC was applied before liver ischemia and was set for six cycles of 4-min ischemia and 4-min reperfusion. And the results showed LRIpreC-induced HO1 expression resulted in autophagy and the alleviation of liver I/R injury.

Another team, Wang et al., used SD rats to understand the detrimental role of high-mobility group box 1 (HMGB1) in cerebral ischemia and how the combination of LRIperC and cerebral ischemic postconditioning can attenuate HMGB1 (95). HMGB1 is a protein secreted by immune cells as a cytokine mediator of inflammation. Thus, this mode of action potentiates its role in inflammation poststroke. Su et al. also used SD rats to perform MCAO to understand the role of LRIperC in conferring neuroprotection (95). LRIperC was performed by four cycles of 10-min ischemia and 10-min reperfusion of the bilateral femoral arteries. Their results indicated that autophagy activation contributed to neuroprotection of LRIperC.

Another study, done by Han et al., used C57BL/6 mice to create myocardial I/R injury model to show the role of LC3-II/LC3-I in autophagy (57). LC3 is a microtubule-associated protein that becomes conjugated during autophagy to form LC3-I and is recruited to autophagosomal membranes (123). Also, Han et al. induced RIC by three cycles of 4-min ischemia and 4-min reperfusion of the left femoral artery (57), and their results showed higher ratios of LC3-II/LC3-I were observed in RIC group after myocardial I/R injury, thus showing involvement of the compound in autophagy. Rohailla et al. used C57BL/6 mice to test the function of RIC to autophagy signaling (60). RIC was performed with four cycles of 5-min ischemia and 5-min reperfusion of the femoral artery. At the conclusion of each experiment, the mouse hearts were dissected for further analyses. They were able to ascertain that RIC was able to induce pro-autophagy signaling. Wang et al., in SD rat models, was able to show that RIC attenuated plasma HMGB1 levels and exerted a neuroprotective effect by inhibiting the autophagy process (51).

Qi and colleagues used SD rats to preform MCAO; LRIP was performed by three cycles of 10-min ischemia and 10-min reperfusion of the bilateral femoral artery at 0, 10 or 30 min after MCA reperfusion (48). Their results showed that AKT/GSK3β-dependent autophagy is very important in LRIP, reducing reperfusion of ischemic brain. In a subsequent study, they were also able to prove that Bcl2 phosphoyrlation and Bcl-2/Beclin 1 complex disruption played a key role in eliciting autophagy and diminishing mitochondrial damage in RIC rats after cerebral ischemia; this required the involvement of the AKT/GSK3β-dependent pathway acitvation (76). Zhou et al. used a hypoxia–ischemia model in which rat pups were induced at postnatal day 10 (73). LRIP was induced directly after hypoxia by four cycles of 10-min hind limb ischemia. LRIP reduced infarct volume at 48 h and enhanced functional outcomes four weeks after hypoxia–ischemia. This was achieved by involving initiation of the opioid receptor/PI3K/AKT signaling pathway. Thus, their group was also able to show the involvement of the AKT/GSK3β-dependent pathway in LRIP and how activation can reduce the damage caused by I/R.

### Transient Receptor Potential Vanilloid 1

Transient Receptor Potential Vanilloid 1 (TRPV1) is a nonselective cation channel expressed in primary sensory nerves that becomes activated from physical/chemical stimuli and releases neuropeptides, calcitonin gene-related protein (CGRP), and substance P (SP). Gao et al. used male SD rats to effectively exhibit reduction in cardiac I/R injury by using LRIP (79). Specifically, they studied the presence or absence of TRPV1 receptor antagonist capsazepine, CGRP receptor antagonist CGRP8-37, or SP receptor antagonist RP-67580. Using these compounds, they were able to show that postconditioning reduced the size of myocardial infarction from I/R injury in which TRPV1 played a significant role. They were also able to show that CGRP and SP are upregulated when TRPV1 receives signals by LRIP and subsequently acts on the corresponding receptors in the heart to reduce infarction size. In 2017, Randhawa and Jaggi used Wistar albino rats to perform LRIpreC and subsequently induced retrograde heart perfusion. Results showed that LRIpreC was able protect the heart by activating TRPV1 channels (89). Hence, these promising results can be applied to cerebral models and further translated to clinical studies.

### Tumor Necrosis Factor α

Tumor Necrosis Factor α is an important index in organ injury. RIC is able to exert protectictive function by decreasing TNFα. Ramagiri and Taliyan used rats to induce bilateral common carotid occlusion, LRIP was achieved by three cycles of 10-min ischemia and 10-min reperfusion of bilateral femoral artery. Results showed that LRIP was able to decrease the level of TNFα (124). Kim et al., using LPS-induced septic mice, initiated LRIpreC by inducing three cycles of 10-min ischemia followed by 10-min reperfusion of the right hind limbs, and the results showed that LRIpreC was able to increase the survival rate and decrease TNFα level (109). Zheng et al. proved that RIC was able to protect against acute acetaminophen-induced liver injury by decreasing serum levels of TNFα in mice (108). Czigany et al. used Wistar rats with induced liver ischemia and found that four cycles of remote ischemic preconditioning were able to decrease TNFα levels and protect the liver (125).

### Adenosine

Adenosine is produced in the body in response to high stress conditions such as inflammation and I/R. Adenosine receptors consist of four G protein-coupled receptors through which it exerts protective effects through A_1_R, A_2A_R, A_2B_R, and A_3_R. Tsubota et al. studied adenosine receptors in the setting of I/R injury and specifically looked at A_1_R and A_2A_R (56). Adenosine and adenosine receptors are important in the protection of RIC in brain or heart ischemic injury. Surendra et al. proved that LRIpreC and LRIP were able to exert myocardial protection by adenosine receptors (126). An et al. used SD rats, operating three cycles of 5-min ischemia and 5-min reperfusion every other day until weeks 4, 6, and 8 after myocardial infarction (127). Results showed that LRIP improved cardiac hemodynamic function by increasing myocardial levels of mitochondrial adenosine triphosphate (127, 128). Researchers also studied the functions of other indexes, such as bradykinin and neuroglobin, in the protection of RIC. Gross et al. proved that abdominal surgical incision causes LRIpreC of trauma by activation of bradykinin receptors (129). Ren et al. induced MCAO in SD rats where LRIpreC was performed in combination with LRIP; they showed that the combination of LRIpreC and LRIP was able to increase the expression of neuroglobin and induce brain damage (130). Our team has also proved that prostaglandin F2α FP receptor antagonist plays an important role in protecting the brain during a TBI. However, until now no research has proved the function of this receptor in RIC (131, 132).

### Prostaglandins

Prostaglandins in both preclinical and clinical studies have been shown to have numerous cytoprotective benefits in both acute and chronic neurological conditions. It was shown in previous studies that prostaglandin PGF_2α_ FP receptor’s presence in the CNS was related to stroke and Ca^2+^ signaling. In our previous studies, we showed that the FP receptor reduced infarct volume in a transient MCAO mouse model. In a subsequent study, we used FP antagonist AL-8810 and FP receptor knockout mice, both *in vivo* and *in vitro*, to show that the inhibitor of the FP receptor improved the outcome in mice after ischemia in relation to neurobehavorial function and infarct volume (133). At this time, there have been very few studies done in ischemic conditioning with prostaglandins. Given the potential for therapeutic options, this is an area that needs to be expanded on.

### Endocannabinoids

Endocannabinoids have been shown to provide protective effects of ischemic preconditioning through cannabinoid CB_1_/CB_2_ receptors. Previous studies on this topic have shown that CB_1_ receptor agonists in knockout mice conferred protection against cerebral I/R injury. Expression of CB_2_ showed decreased ROS formation, inflammatory cell chemotaxis, and inflammatory cell activation; expression of CB_1_ showed decreased core body temperature, increased neuroprotective signaling, and increased coronary and cerebral dilation (134). The exact mechanism of this neuroprotection is not well understood yet; however, multiple preclinical studies have shown reduced infarct size and decreased motor disability poststroke. Leker et al. used a CB1 agonist, HU-210, on rats that underwent permanent MCAO to show significantly reduced motor disability and infarct volumes (135). Their team used a standardized motor disability scale and showed significant improved outcomes in rats that were pretreated with HU-210. Thus, these preliminary preclinical studies have shown that endocannabinoids can confer neuroprotection in cerebral ischemia. Further studies and research can expand on potential clinical studies and eventual therepeutic options.

### Neuroglobin

Neuroglobin (Ngb) is an intracellular hemoprotein that is expressed in the CNS and CSF and reversibly binds to oxygen with an affinity higher than that of hemoglobin. During ischemic injury, neuroglobin increases oxygen availability in the brain to limit the extent of infarction. Thus, Ngb has been studied in LRIperC and LRIP models to show attenuation of I/R injury. Ren et al., in their study with rats, subjected them to MCAO; limb perconditioning was immediately applied followed by repeated, short episodes of remote ischemia 24 h after reperfusion (130). Their team found that ischemic per- and postconditioning increased expression of Ngb. They were able to show that this process upregulated Ngb, which is a known neuroprotectant in the setting of stroke. Further research will facilitate the production of therapuetic agents and the use of them in both preclinical and clinical trials.

## Potential Issues of Anesthestics Used in Preclinical Studies

### Chloral Hydrate

Researches have demonstrated that chloral hydrate confers protection to cardiovascular and cerebral I/R injury. Liu et al. used male C57BL/6J mice or ANXA1 knockout mice to induce MCAO 1 h prior to RIC (136). The chloral hydrate concentrations of 2, 6, and 10% were injected intraperitoneally to different groups. Their results indicated that chloral hydrate preconditioning offered protection against ischemic injuries. This effect was seen through the upregulation of the expression of ANXA1. However, it is difficult to determine if the anesthetic used actually provided a positive influence in the presence of other confounding variables. Nevertheless, several researchers have used chloral hydrate to anesthetize rats or mice for MCAO surgery with promising results (50, 137, 138). Hence, it is important to continue studies with chloral hydrate to isolate its benefits in relation to I/R injury.

### Isoflurane

Isoflurane is widely used for rat or mouse MCAO surgery. Several researchers showed that isoflurane was able to shield the heart and brain from ischemic damage (139–141). On the other hand, some researchers proved that isoflurane had no protection in the ischemic model. For example, Toner et al. used Wistar rats to make brain slices to induce ischemia *in vitro* (142). They found that isoflurane had no protective effect on the ischemic brain slice model. Ruta et al. used SD rats to induce MCAO (143). They also concluded isoflurane provided substantial benefit. Of note, multiple experiments utilized 5% isolfurane. However, this concentration would cause the anesthestic to linger and obscure the outcome data as it can interfere with testing. Thus, it is imperative to be mindful of the dosing of anesthetic used so that it does not produce dubious results.

### Ketamine

Ketamine is widely used in anesthesia and certain rat models. Xue et al. and Mathews et al. used rat cerebral cortical slices to induce an oxygen-glucose deprived state; results showed that ketamine provided neuroprotective effects (144, 145). However, Todd et al. used rat an MCAO model to show that ketamine had no protective benefits on their model (146). These contrasting results between the two study groups may be a result of differing concentrations and durations of the anesthestic used. Hence, further research is required to examine the potential benefits of ketamine on limb RIC.

## Conclusion and Perspectives

The LRIpreC paradigm was first described in 1986; however, the potential for clinical translation has only been realized in the past 5–10 years (147). RIC, in its diverse forms (LRIpreC, LRIperC, and LRIP), signals the potential of a robust, high-fidelity, inexpensive, and accessible path to organ protection in the clinical setting (148). Two main reasons come to mind when considering why it has been difficult to translate the cerebroprotective effects of ischemic conditioning from preclinical to clinical studies. First, there has been an inadequacy of animal models. More specifically, the models have been limited to young, male mice. There has been no evidence offered that RIC is effective in aged rodents and only some evidence of its effectiveness is seen in females (49, 59, 78). Actually, in clinical studies, RIC would be used to treat aged persons and persons with comorbidities, such as hypertension, diabetes, and dyslipidemia. Also, the use of RIC would not be limited only to males, as it is in preclinical models at this time.

**Figure 2:**
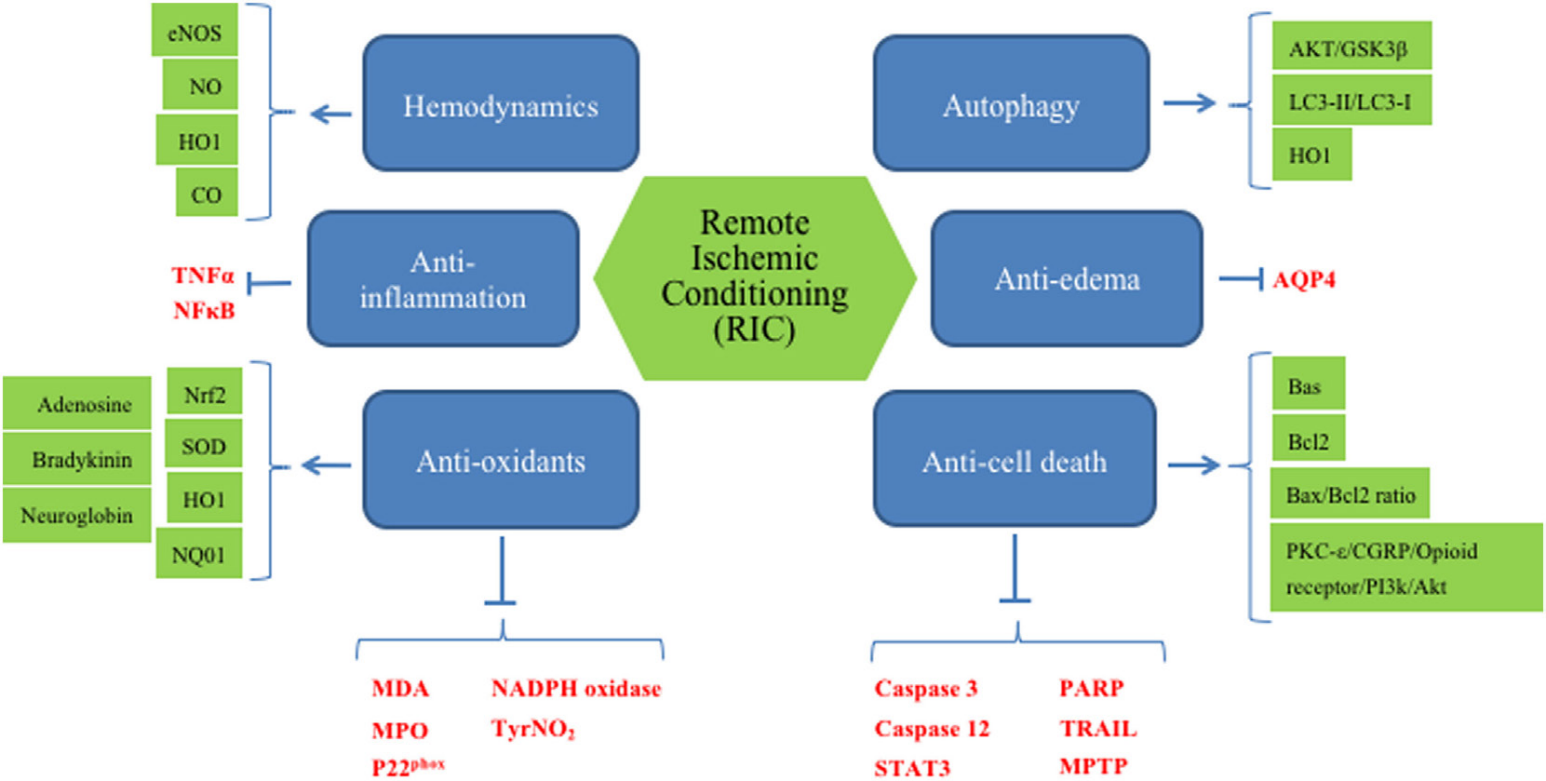
Overview of presumably all mechanisms, known to date, involved in remote ischemic conditioning (RIC).

Second, RIC will be performed on patients who will be on other medications, such as circulating plasminogen activators, anti-hypertensives, hypoglycemic agents, lipid lowering agents, and many more. Thus, it is difficult to assess the effect of RIC when there are other confounding factors involved. However, detailing the cellular and systemic pathways, as we have done in Figure 2, and identifying potential biomarkers in preclinical studies would facilitate that translation to clinical use.

The importance of biomarkers is to gauge the conditioning response in humans. Presumptive biomarkers include adenosine, bradykinin, endogenous opioids, anti-inflammatory, proinflammatory cytokines, NO, and nitrite. Measuring these could assist in confirming that a threshold for a conditioning response has been met. Studying preclinical models in parallel with clinical models can help understand pathways more succinctly and assist with the translation to clinical practice.

For the operational methods of RIC, one important variable that must be explored is the time and duration of each cycle. Table 1 shows that the popular operational methods for RIC are three cycles of 5-min distal organ I/R for one session per day and three or four cycles of 10-min distal organ I/R for one session per day (50, 53, 62, 77). Until now, there have been limited results that proved exactly how many cycles would be necessary for maximum effectiveness. Knowing the minimal effective cycle number would benefit in the clinical setting as we can keep the exposure of patients to RIC at a minimum to limit side effects and potentially harmful sequelae.

This article, to our knowledge, is the first extensive literature review of the various mechanisms involved in RIC studied in the preclinical models to date. As an attractive low-cost, low-risk therapy, the application of LRIP and LRIperC can be utilized in a wide range of clinical settings such as cerebral, myocardial, and hepatic I/R injury. Clinical uncertainties should be taken into account to give meaning and value to the experimental studies that will be performed. This will help us translate studies from preclinical into clinical and eventually help us implement routine procedures in the hospital setting.

## Author Contributions

SD designed the manuscript; GC, MT, CR, and SD wrote the manuscript and agreed on the final version.

## Conflict of Interest Statement

The authors declare that the research was conducted in the absence of any commercial or financial relationships that could be construed as a potential conflict of interest.

## Abbreviations

AQP4, aquaporin-4; Bas, biotrophy-associated secreted protein; BAX, Bcl2 associated X protein; Bcl2, B-cell leukemia/lymphoma 2; eNOS, endothelial nitric oxide synthase; HMGB1, high mobility group box 1; HO1, heme oxygenase 1; MDA, malondialdehyde; MMP-9, matrix metallopeptidase 9; NFκB, nuclear factor kappa B; NO, nitric oxide; NQO1, NAD(P)H quinone oxidoreductase 1; Nrf2, nuclear factor erythroid-derived 2-related factor 2; PARP, poly (ADP-ribose) polymerase; SOD, superoxide dismutase; STAT3, signal transducer and activator of transcription 3; TNFα, tumor necrosis factor α; TRAIL: tumor necrosis factor-related apoptosis-inducing ligand.
